# Dr. John S. Clark

**Published:** 1867-05

**Authors:** 


*Obituary.*—Dr. John S. Clark, formerly of New Orleans, died on the 29th of November, 1866, at his residence in St. Louis, where he had been in practice for several years. Dr. Clark fully illustrated the rare combination of high moral and social virtues with eminent professional acquirements. No higher evidence could be afforded of his professional and social standing than is expressed in the appropriate resolutions passed by the Dentists of St. Louis, at a meeting called upon the announcement of his death.

We have also to announce the death of Dr. A. M. Leslie, formerly of Cincinnati, whose name is familiar to our readers from his connection with the Miss. Valley Dental Depot. His thorough knowledge of the wants of his professional brethren rendered him peculiarly qualified for the calling in which he had been engaged for a number of years past, in Cincinnati, St. Louis, and Memphis.

John R. McCurdy, Esq., whose name is also familiar to the dental profession, from his connection with the old firm of Jones, White & McCurdy, died n Philadelphia, in February last.

